# Workplace Accommodations and Attrition Among Physicians With Disabilities

**DOI:** 10.1001/jamanetworkopen.2026.1922

**Published:** 2026-03-23

**Authors:** Zoie C. Sheets, Zakia Nouri, Sarah S. Conrad, Christopher J. Moreland, Xiaochu Hu, Michael J. Dill, Lisa M. Meeks

**Affiliations:** 1 Department of Internal Medicine, University of Chicago, Chicago, Illinois; 2 Department of Pediatrics, University of Chicago, Chicago, Illinois; 3 Association of American Medical Colleges, Washington, DC; 4 Department of Internal Medicine, Dell Medical School, University of Texas at Austin, Austin; 5 Department of Medicine Education, University of Illinois at Chicago, Chicago; 6 Department of Family Medicine, University of Michigan Medical School, Ann Arbor

## Abstract

**Importance:**

Physicians with disabilities face bias and barriers in the workplace, including stigma, lack of accommodations, and mistreatment, which may contribute to workforce attrition. Given the projected physician shortage and the importance of physicians with disabilities in providing informed and empathetic care, understanding attrition within this group is critical.

**Objective:**

To examine the associations among disability, workplace accommodations, and physician workforce attrition, including consideration of leaving medical practice and reductions in clinical hours.

**Design, Setting, and Participants:**

This survey study used a cross-sectional design to analyze data from the 2022 National Sample Survey of Physicians. Logistic regression models assessed associations between disability and attrition outcomes, adjusting for demographic and workplace factors. Participants included 5917 active physicians who self-reported personal (eg, disability status) and professional (eg, accommodations) data. Data were collected from May 10 to November 9, 2022, and analyzed from October 1, 2023, to May 1, 2025.

**Main Outcomes and Measures:**

The primary outcomes were (1) having considered leaving medical practice within the past 12 months, including reasons why, and (2) having ever reduced clinical hours for 6 months or longer. The core independent variable was accommodation status.

**Results:**

Among the 5917 physicians surveyed, 154 (2.6%) reported having a disability. A total of 3707 respondents (62.6%) were men or transgender men and 5620 (95.0%) identified as heterosexual; the mean (SD) age was 53.9 (10.8) years. Fifty-six physicians with disabilities (36.4%) considered leaving the practice of medicine, compared with 1316 of 5600 physicians (23.5%) without disabilities. Sixty-seven physicians with disabilities (43.5%) reported transitioning to part time or pausing their practice at some point, compared with 1327 (23.7%) without disabilities. Multivariate regression analysis found physicians with disabilities were more likely than their peers without disabilities to consider leaving medical practice (odds ratio [OR], 2.22; 95% CI, 1.24-3.96; *P* = .01) and to have reduced clinical hours or paused practice during their careers (OR, 1.94; 95% CI, 1.09-3.43; *P* = .02). Burnout was the most common reason among both groups, and physicians with disabilities more frequently cited underlying health conditions (self or family) (32 [52.7%] vs 122 [8.5%]). Among physicians with disabilities, those who received accommodations were significantly less likely than those without accommodations to report an intent to leave (42 of 123 [34.3%] and 13 of 24 [54.2%], respectively).

**Conclusions and Relevance:**

In this survey study, physicians with disabilities were significantly more likely to consider leaving the workforce and to reduce clinical hours than their peers without disabilities. Clear, stigma-free disclosure and accommodation processes, along with inclusive workplace cultures, are essential to retaining this vital segment of the physician workforce.

## Key Points

**Question:**

Are physicians with disabilities more likely to leave the workforce or reduce practice, and how many of these physicians receive accommodations?

**Findings:**

In this survey study of 5917 US physicians, respondents with disabilities were more than twice as likely to consider leaving medicine and nearly twice as likely to reduce or pause clinical practice compared with their peers without disabilities. Among physicians with disabilities, 80% reported receiving accommodations.

**Meaning:**

These findings suggest that physicians with disabilities are at greater risk of workforce attrition due to burnout, health conditions, and unsafe work environments; accommodations may reduce this risk.

## Teaser

This survey study examines the association between physician disability and the intent of those physicians to leave the workforce or reduce participation in medical practice.

## Introduction

The US health care system is projected to face a shortage of 13 500 to 86 000 physicians by 2036, which may be exacerbated by physicians leaving or pausing clinical practice.^1,2^ This workforce attrition is likely to impact marginalized patient populations, including individuals with disabilities, disproportionately.^3^ Physicians with disabilities are an underrepresented yet essential part of the health care workforce,^4^ offering informed and empathetic perspectives on patient care. Patients with disabilities often experience bias, and increasing the number of physicians with lived experience of disability may improve their care.^5,6^ Given this and the extant shortage of physicians, it is imperative to understand the factors driving workforce reductions among physicians with disabilities and the reasons influencing their decisions to leave or pause clinical practice.

Research has identified mistreatment, depression, and burnout as key drivers of health care workforce attrition.^7,8^ Burnout, in particular, has profound implications for patient care and is associated with increased patient safety incidents.^7^ Physicians with disabilities experience disproportionately high rates of burnout and mistreatment,^9,10^ placing them at heightened risk of leaving the workforce.

Accommodations may play a critical role in supporting physicians with disabilities. Unfortunately, physician trainees often struggle to obtain accommodations.^11,12,13^ Fear of stigma or bias, unclear institutional processes, and uncertainty and fear surrounding accommodations for learners and leaders may contribute to these struggles.^12,13^ Almost 50% of resident physicians with disabilities lack accommodations, leading to serious consequences, including higher rates of depressive symptoms and medical errors, compared with their peers without disabilities.^11^ This problem is not witnessed when accommodations are provided.^11^

Despite evidence linking burnout and mistreatment to workforce attrition,^7,8^ and known burnout and mistreatment of physicians with disabilities,^9,10^ intent to leave or reduce clinical practice and accommodation status among physicians with disabilities remain underexplored. This study reports workplace accommodation status among physicians with disabilities. It fills a critical gap by examining the association among disability, receipt of accommodation, and intent to leave or reduce participation in medical practice among physicians with disabilities.

## Methods

### Data Source

In this survey study using a cross-sectional design, we analyzed the Association of American Medical Colleges’ 2022 National Sample Survey of Physicians (NSSP) data. The NSSP, collected from May 10 to November 9, 2022, contains information on 5917 nationally representative, active physicians. The Association of American Medical Colleges provides details about NSSP sampling and weighting strategies on its website.^13^ NSSP respondents reported whether they had any disability and the type of disability, whether they received disability accommodations in their workplace, and whether they considered leaving medical practice during the last 12 months, reduced their hours to part time, or completely paused practice for at least 6 months during their career and reasons for doing so (eAppendix in Supplement 1). This survey study followed the American Association for Public Opinion Research (AAPOR) reporting guideline and was deemed exempt from review and informed consent by the American Institutes for Research Institutional Review Board.

### Statistical Analysis

Data were analyzed from October 1, 2023, to May 1, 2025. We conducted 2 logistic regressions to investigate the association between physicians’ disability status and self-reported attrition measures, including considering leaving the practice of medicine in the past year and temporarily reducing clinical practice hours (transitioning to part time or pausing completely) for at least 6 months during their practice history. Those who did not respond or who responded, “I don’t know” to disability status were excluded from this analysis.

The models included covariates such as physicians’ demographic factors (age, gender, sexual orientation, marital and parental status, and race and ethnicity), workplace characteristics (work settings, weekly work hours, specialty group, academic affiliation), and US or International Medical Graduate status. Self-identified race and ethnicity were stratified as American Indian or Alaska Native; Asian; Black or African American; Hispanic, Latino, or of Spanish origin; Native Hawaiian or Other Pacific Islander; White; multiracial or other (including Middle Eastern or other); and missing. These data were included in this study to separate the effects of race and ethnicity from those of disability status on decisions to leave the workforce or alter work hours. Prior research on physicians with disabilities substantiates this approach.^9,10^ Two-sided *P* < .05 indicated statistical significance. Statistical analyses were conducted using Stata, version 18.0 (StataCorp LLC).

## Results

Among surveyed physicians (n = 5917), 154 (2.6%) reported having a disability, 5600 (94.6%) reported having no disability, and 163 (2.8%) did not disclose their disability status (Table 1). The mean (SD) age was 53.9 (10.8) years. A total of 2195 respondents (37.1%) identified as women or transgender women, 3707 (62.6%) identified as men or transgender men, and 16 (0.3%) identified as genderqueer or other. In terms of sexual orientation, 5620 respondents (95.0%) identified as heterosexual or straight, 104 (1.8%) identified as gay or lesbian, and 144 (2.48%) identified as other. In terms of race and ethnicity, 7 respondents (0.1%) were American Indian or Alaska Native, 1504 (25.4%) were Asian, 14 (0.2%) were Black, 149 (2.5%) were Hispanic, 13 (0.2%) were Native Hawaiian or Other Pacific Islander, 3838 (64.9%) were White, 263 (4.4%) were multiracial or of other race or ethnicity, and 16 (0.3%) had missing data.

**Table 1.** Demographic and Work Characteristics of Participating Physicians

Characteristic	Respondent group, No. (%)^a^			
	Physicians without disabilities (n = 5600)	Physicians with disabilities (n = 154)	Missing disability status (n = 163)	All (N = 5917)
Age, mean (SD), y	53.8 (10.8)	59.9 (9.8)	52.4 (9.9)	53.9 (10.8)
Age group, y				
<41	675 (98.0)	3 (0.4)	11 (1.6)	689 (11.6)
41-50	1771 (94.5)	30 (1.6)	73 (3.9)	1874 (31.7)
51-60	1406 (94.7)	37 (2.5)	41 (2.8)	1484 (25.1)
61-70	1434 (93.9)	62 (4.1)	31 (2.0)	1527 (25.8)
≥71	314 (91.3)	22 (6.4)	8 (2.3)	344 (5.8)
Gender identity				
Women and transgender women	2056 (93.7)	72 (3.3)	67 (3.0)	2195 (37.1)
Men and transgender men	3531 (95.3)	79 (2.1)	97 (2.6)	3707 (62.6)
Genderqueer or other	13 (81.3)	3 (18.8)	0	16 (0.3)
Race and ethnicity				
American Indian or Alaskan Native	4 (57.1)	0	3 (42.9)	7 (0.1)
Asian	1428 (94.9)	21 (1.4)	55 (3.7)	1504 (25.4)
Black or African American	126 (98.4)	2 (1.6)	0	128 (2.2)
Hispanic, Latino, or of Spanish origin	140 (94.0)	5 (3.4)	4 (2.7)	149 (2.5)
Native Hawaiian or Other Pacific Islander	8 (61.5)	0 (	5 (38.5)	13 (0.2)
White	3649 (95.1)	112 (2.9)	77 (2.0)	3838 (64.9)
Multiracial or other^b^	231 (87.8)	13 (4.9)	19 (7.2)	263 (4.4)
Missing	14 (87.5)	2 (12.5)	0	16 (0.3)
Sexual orientation				
Heterosexual or straight	5332 (94.9)	143 (2.5)	145 (2.6)	5620 (95.0)
Other	268 (90.1)	11 (3.9)	18 (6.0)	297 (5.0)
Married				
No	857 (92.2)	39 (4.2)	33 (3.6)	929 (15.7)
Yes	4742 (95.1)	115 (2.3)	130 (2.6)	4987 (84.3)
Presence of children aged ≤5 y				
No	4900 (94.4)	151 (2.9)	142 (2.7)	5193 (87.8)
Yes	699 (96.7)	3 (0.4)	21 (2.9)	723 (12.2)
Working in hospitals	1322 (95.6)	32 (2.3)	29 (2.1)	1383 (23.4)
Specialty group				
Medical specialties	973 (94.2)	26 (2.5)	34 (3.3)	1033 (17.5)
Other	1868 (94.4)	51 (2.6)	59 (3.0)	1978 (33.4)
Primary care	1724 (94.2)	58 (3.2)	48 (2.6)	1830 (30.9)
Surgery	1034 (96.1)	20 (1.9)	22 (2.0)	1076 (18.2)
International medical graduate	1045 (94.4)	23 (2.1)	39 (3.5)	1107 (18.7)
Academically affiliated	1988 (95.7)	58 (2.8)	32 (1.5)	2078 (35.1)
Accommodations provided	NA	130 (100)	NA	130 (2.2)

Abbreviation: NA, not applicable.

^a^ Counts were weighted and rounded to the nearest integer. Percentages were calculated based on weighted, nonrounded counts. Data are from the Association of American Medical Colleges 2022 National Sample Survey of Physicians. Disability is self-reported. Those who responded “I don’t know” were excluded from the analyses. Percentages for the 3 subgroups are calculated by row; percentages for all respondents, by column.

^b^ Includes Middle Eastern and other.

### Intent to Leave Practice

Among physicians with disabilities, 56 of 154 (36.4%) reported an intention to leave the practice of medicine, compared with 1316 of 5600 (23.5%) without disabilities. When accounting for the covariates, physicians with disabilities had higher odds of considering leaving the practice of medicine in the past year for reasons other than planned retirement compared with physicians without disabilities (odds ratio [OR], 2.22; 95% CI, 1.24-3.96; *P* = .01) (Table 2).

**Table 2.** Intention to Leave and Reduce Clinical Hours by Demographic and Work Characteristics

Characteristic	Intention to leave (n = 5610)		Decrease hours or pause clinical practice (n = 5639)	
	AOR (95% CI)	*P* value	AOR (95% CI)	*P* value
Physicians without disabilities	1 [Reference]	NA	1 [Reference]	NA
Physicians with disabilities	2.22 (1.24-3.96)	.01	1.94 (1.10-3.43)	.02
Gender identity				
Woman and transgender woman	1 [Reference]	NA	1 [Reference]	NA
Man and transgender man	0.72 (0.57-0.90)	.01	0.34 (0.27-0.43)	<.001
Genderqueer or other	5.46 (1.52-19.65)	.01	0.33 (0.05-2.10)	.24
Race and ethnicity				
American Indian or Alaskan Native	0.19 (0.03-1.09)	.06	0.11 (0.01-1.16)	.07
Asian	1.01 (0.52-1.94)	.99	1.10 (0.54-2.27)	.79
Black or African American	1.19 (0.46-3.04)	.72	2.79 (1.16-6.75)	.02
Hispanic, Latino, or of Spanish origin	1 [Reference]	NA	1 [Reference]	NA
Native Hawaiian or Other Pacific Islander	0.12 (0.02-0.60)	.01	0.08 (0.01-0.44)	.004
White	1.04 (0.55-1.97)	.91	1.031 (0.52-2.06)	.93
Multiracial or other^a^	1.24 (0.56-2.73)	.60	1.39 (0.57-3.41)	.47
Sexual orientation				
Heterosexual	1.10 (0.66-1.85)	.71	1.39 (0.75-2.59)	.30
Other	1 [Reference]	NA	1 [Reference]	NA
Age group, y				
<41	1 [Reference]	NA	1 [Reference]	NA
41-50	1.06 (0.72-1.56)	.78	0.90 (0.58-1.42)	.66
51-60	0.70 (0.46-1.06)	.09	1.07 (0.67-1.71)	.76
61-70	0.42 (0.27-0.64)	<.001	1.56 (0.98-2.47)	.06
≥71	0.29 (0.14-0.56)	<.001	2.87 (1.66-4.97)	<.001
Married	0.89 (0.66-1.20)	.46	0.99 (0.74-1.33)	.95
Presence of children aged ≤5 y	0.85 (0.58-1.25)	.42	1.01 (0.65-1.53)	.98
Working in hospitals	1.13 (0.86-1.48)	.39	0.83 (0.62-1.12)	.23
Specialty group				
Medical specialties	1 [Reference]	NA	1 [Reference]	NA
Other	1.33 (0.97-1.81)	.08	1.25 (0.90-1.73)	.18
Primary care	1.13 (0.83-1.52)	.44	1.25 (0.92-1.70)	.16
Surgery	0.90 (0.63-1.28)	.54	0.81 (0.56-1.18)	.28
Usual hours of work in a week	1.007 (1.000-1.014)	.05	0.97 (0.97-0.98)	<.001
Academically affiliated	0.92 (0.73-1.17)	.49	0.91 (0.72-1.12)	.46
International medical graduate	0.65 (0.48-0.90)	.01	0.58 (0.41-0.82)	.002

Abbreviations: AOR, adjusted odds ratio; NA, not applicable.

^a^ Includes Middle Eastern or other.

Burnout was the most frequently cited reason for considering leaving or pausing practice among physicians with and without disabilities (41 of 61 [67.5%] vs 1104 of 1433 [77.0%]) (Figure, A). For physicians with disabilities, burnout was followed by underlying health conditions (self or family) (32 of 61 [52.7%] vs 122 of 1433 [8.5%]) and high-risk working conditions (15 of 61 [24.5%] vs 381 of 1433 [26.6%]). Among physicians without disabilities, burnout (1104 of 1433 [77.0%]) was followed by high-risk working conditions (381 of 1433 [26.6%]) and workforce shortages (337 of 1433 [23.5%]). Among physicians with disabilities, 123 (79.9%) reported receiving accommodations from their employers and 31 (20.1%) did not. In a subsequent question about why their employer did not provide reasonable accommodations, 7 people responded, “I have not requested accommodations because I feel I do not need accommodations.” We excluded these 7 individuals from the following analysis. Of those who received accommodations for their disabilities, 42 of 123 (34.3%) considered leaving medicine. In contrast, 13 of 24 physicians with disabilities (54.2%) who did not receive needed accommodations expressed similar intentions.

**Figure.**
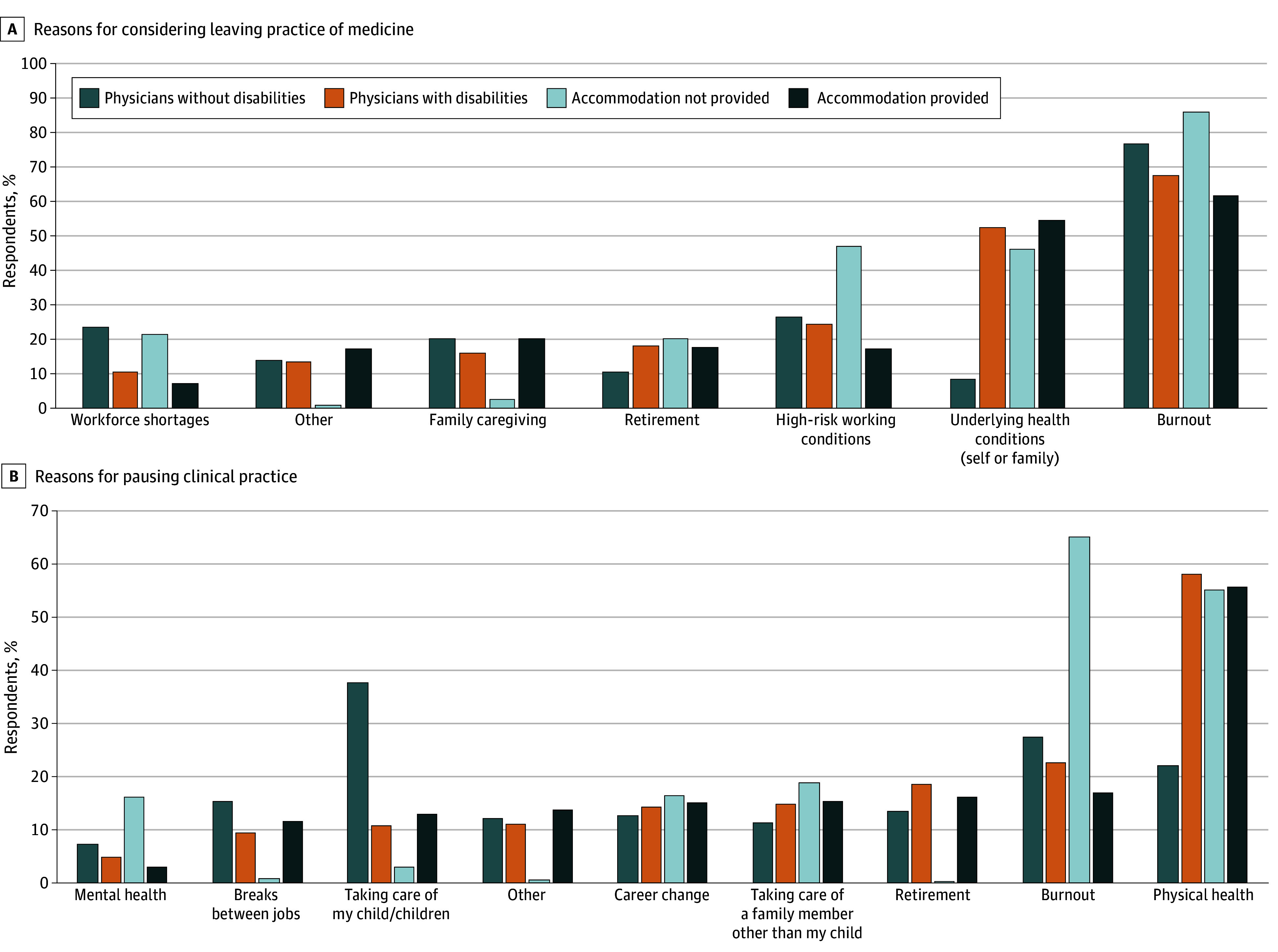
Bar Graphs Showing Reasons for Considering Leaving or Pausing Clinical Practice by Disability and Accommodations Status Percentages do not sum to 100 because respondents could select multiple applicable responses.

### Reducing or Pausing Practice

Sixty-seven physicians with disabilities (43.5%) reported transitioning to part time or pausing their practice at some point, compared with 1327 physicians without disabilities (23.7%). Regression analyses indicated that when compared with physicians without disabilities, those with disabilities were significantly more likely to transition to part time or pause their clinical practice entirely (OR, 1.94; 95% CI, 1.09-3.43; *P* = .02) (Table 2). Physical health concerns (36 of 62 [58.1%] vs 272 of 1231 [22.1%]), burnout (14 of 62 [22.6%] vs 339 of 1231 [27.5%]), and retirement (12 of 62 [18.5%] vs 164 of 1231 [13.4%]) were the most cited reasons among physicians with disabilities who reduced their clinical hours or paused their practice compared with their peers without disabilities (Figure, B).

## Discussion

Physicians with disabilities were more likely to reduce their clinical hours or consider leaving medicine than their peers without disabilities. Furthermore, a higher proportion of physicians with disabilities who did not receive accommodations considered leaving medical practice than those who did. These findings highlight the critical role of workplace accommodations in retaining physicians with disabilities.

In keeping with current literature,^4^ burnout was the most frequently cited reason for leaving the workforce among all participants. Notably, for those without accommodations, the rate at which physicians cite burnout as a driver to leave or pause clinical practice was higher than for other reasons. These findings suggest that access to accommodations for physicians with disabilities may be critical to workforce retention and mitigating burnout.

A clear process for requesting accommodations and transparency about the process are critical to improving access to accommodations and reducing the stigma that may prevent disclosure.^13,14^ Given the known association between mistreatment and burnout, institutions must establish clear policies and reporting mechanisms prohibiting and responding to mistreatment.^2,8^

This study provides novel information on the association between disability and accommodation status and considering leaving the workforce. Our nationally representative dataset enhances the generalizability of the findings. By including multiple measures of attrition—intent to leave and reduction or pause in clinical hours—we provide a robust view of workforce retention challenges.

### Limitations

This study has limitations. Since participants were active physicians and our measures were intention to leave and pause practice, we may not have captured physicians who left the workforce permanently. The survey design also did not permit us to distinguish between reductions in practice hours as proactive accommodations vs a consequence of unmet access needs. Additionally, self-reported disability data may be influenced by fear of disclosure or self-identification with the word *disabled*. As a result, the estimate of how many physicians with disabilities are leaving the workforce may understate the true magnitude of the issue. Outcomes may have also been impacted by the proximity of this survey to the COVID-19 pandemic. Finally, the survey item *underlying health conditions* included those of self or family. This limits our understanding of whether the personal health conditions of physicians with disabilities vs caregiver responsibilities may contribute to intention to leave.

## Conclusions

This survey study using a cross-sectional design found that physicians with disabilities, particularly those without accommodations, were more likely to leave the workforce or pause practice than their peers without disabilities. Addressing these issues is crucial for retaining this valuable segment of the workforce. Health systems should prioritize robust, confidential processes for requesting accommodations, enforce zero-tolerance policies on harassment and bias, and include disability as a core component of diversity initiatives.

## Notes

Supplement 1. **eAppendix.** National Sample Survey of Physicians (NSSP) Questions

Supplement 2.
Data Sharing Statement
