# Supplementary material for: Workplace Accommodations and Attrition Among Physicians With Disabilities
Source: JAMA Netw Open. 2026 Mar 23;9(3):e261922. doi: 10.1001/jamanetworkopen.2026.1922 (PMC13010203; doi:10.1001/jamanetworkopen.2026.1922)
Supplement: Supplement 1. — eAppendix. National Sample Survey of Physicians (NSSP) Questions [file jamanetwopen-e261922-s001.pdf]

Supplementary Online Content

Sheets ZC, Nouri Z, Conrad SS, et al. Workplace accommodations and attrition among physicians with disabilities. *JAMA Netw Open.* 2026;9(3):e261922. doi:10.1001/jamanetworkopen.2026.1922

eAppendix. National Sample Survey of Physicians (NSSP) Questions

This supplementary material has been provided by the authors to give readers additional information about their work.

© 2026 Sheets ZC et al. *JAMA Network Open*.

eAppendix. National Sample Survey of Physicians (NSSP) Questions

**Disability Related Questions**
**Indication of Disability**
The Americans with Disabilities Act (ADA) defines a person with a disability as a person who has a physical or mental impairment that substantially limits one or more major life activities.<br>Do you have a disability as defined by the ADA?<br>Yes<br>No<br>I don't know
Which of the following best describe your disability? (Please select all that apply.)<br>RANDOMIZE LIST<br>Attention deficit/hyperactivity disorder<br>Chronic health issue<br>Hearing impairment<br>Learning disability<br>Mobility disability<br>Psychological disability<br>Vision impairment<br>Other, please specify ____________________ [ANCHOR]
**Presence/Absence of Reasonable Accommodations**
Has your employer provided reasonable accommodations for your disability?<br>Yes [End disability questions]<br>No [Skip logic to next question]
You indicated that your employer has not provided reasonable accommodation for your disability. Which of the following best describes why your employer did not or has not provided accommodations:<br><br>My request for accommodations was denied<br>My request for accommodations is under review<br>I have not requested accommodations because I feel I do not need accommodations<br>Fear of stigma or bias<br>I do not have documentation to support my request<br>My institution does not have a clear process for requesting accommodation<br>My institution does not have the resources to provide my accommodation needs<br>Other, please specify _______
**Attrition Measures**
**Decreasing clinical hours and reasons why**
Looking back over your entire career as a practicing physician, have you ever temporarily decreased your clinical practice hours (to part-time or paused completely) for at least 6 months? (Please check all that apply.)

© 2026 Sheets ZC et al. *JAMA Network Open*.

No<br>Yes – decreased to part-time<br>Yes – paused clinical practice completely
You indicated that you decreased your clinical practice hours (to part-time or paused completely) for at least 6 months at some point during your career. What were the main reasons you did so? (Please select all that apply.)<br><br>Personal health – physical health<br>Personal health – burnout<br>Personal health – other mental health<br>To care for a family member (other than my child/children)<br>Taking care of my child/children<br>Career change (e.g., full time consulting or administrative position)<br>Break between jobs<br>Intended to retire<br>Other, please specify _______
**Intent to leave the workforce and reasons why**
Have you considered leaving the practice of medicine in the past year (for reasons other than planned retirement)?<br>Yes [Skip logic to next question]<br>No [End line of questioning about intent to leave]
You indicated that you have considered leaving the practice of medicine in the past year (for reasons other than planned retirement). What are the main reasons you have considered leaving the practice of medicine? (Please select all that apply.)<br>Burnout<br>Family caregiving<br>High-risk working conditions<br>Workforce shortages<br>Retirement<br>Underlying health condition(s) (self or family)<br>Other, please specify ________

© 2026 Sheets ZC et al. *JAMA Network Open*.
