# Supplementary material for: Workplace Accommodations and Attrition Among Physicians With Disabilities
Source: JAMA Netw Open. 2026 Mar 23;9(3):e261922. doi: 10.1001/jamanetworkopen.2026.1922 (PMC13010203; doi:10.1001/jamanetworkopen.2026.1922)
Supplement: Supplement 2. — Data Sharing Statement [file jamanetwopen-e261922-s002.pdf]

Data Sharing Statement

Sheets. Workplace Accommodations and Attrition Among Physicians With Disabilities. *JAMA Netw Open*. Published March 23, 2026. doi:10.1001/jamanetworkopen.2026.1922

Data
Data available: Yes
Data types: Deidentified participant data, Data dictionary
How to access data: Only aggregate data and the data dictionary are available in compliance with the IRB unless a data licensing agreement is completed. Individuals interested in the data must complete a request for data through an AAMC data steward, mdill@aamc.org.
When available: With publication

Supporting Documents
Document types: None

Additional Information
Who can access the data: Researchers whose proposed use of the data has been approved.
Types of analyses: Research purposes.
Mechanisms of data availability: with signed data access agreement
